# Linkages between environmental sustainability, disaggregated emission, renewable energy, and energy efficiency: An evidence from BRI countries

**DOI:** 10.1371/journal.pone.0305188

**Published:** 2024-08-21

**Authors:** Fei Meng, Weiyan Sheng, Muhammad Waqas Akbar

**Affiliations:** 1 School of Foreign Languages and Business, and Shenzhen Digital Trade Research Institute, Shenzhen Polytechnic University, Nanshan District, Shenzhen, Guangdong, China; 2 School of Business, Jiangsu Ocean University, Haizhou, Lianyungang, Jiangsu, China; 3 Southern Alberta Institute of Technology, Calgary, Alberta, Canada; Islamia University of Bahawalpur, PAKISTAN

## Abstract

This paper examines the long-term and short-run causative relationship among environmental sustainability, energy efficiency, renewable energy and carbon emissions from all over sources (coal, oil and fossil fuels) and sector wise division (heat and power, transportation, residential, manufacturing and other sectors. The empirical evidence presented in this study is derived from a balanced panel dataset spanning the annual periods from 2000 to 2021. The dataset specifically focuses on a selection of BRI Countries. The Kao test demonstrates the presence of cointegration across variables such as carbon dioxide emissions, environmental suitability, energy efficiency and renewable energy. The Panel Pooled Mean Group-Autoregressive Distributed Lag (PMG-ARDL) model indicates a statistically significant positive association between the environmental sustainability and disaggregated CO_2_ emissions over a long-term period. The study found a positive relationship between disaggregated CO_2_ emissions and environmental sustainability and energy efficiency, with renewable energy sources reducing emissions. It suggests a need for a structural transition from an energy-intensive economy to a decarbonized one, with sectors like heat and power positively impacting sustainability. Implementing measures to reduce emissions is crucial for tackling climate change.

## Introduction

The Belt and Road Initiative (BRI) is a transnational development initiative led by China that aims to enhance connectivity and promoting economic cooperation across the continent of Asia, Europe, and Africa. As the BRI involves extensive infrastructure projects and increased trade activities, there is a growing concern about its potential environmental impact. Understanding the linkages between environmental sustainability, disaggregated emissions, renewable energy, and energy efficiency in BRI countries is crucial for designing effective policies that promote sustainable development within the BRI framework [1].

**Fig 1:**
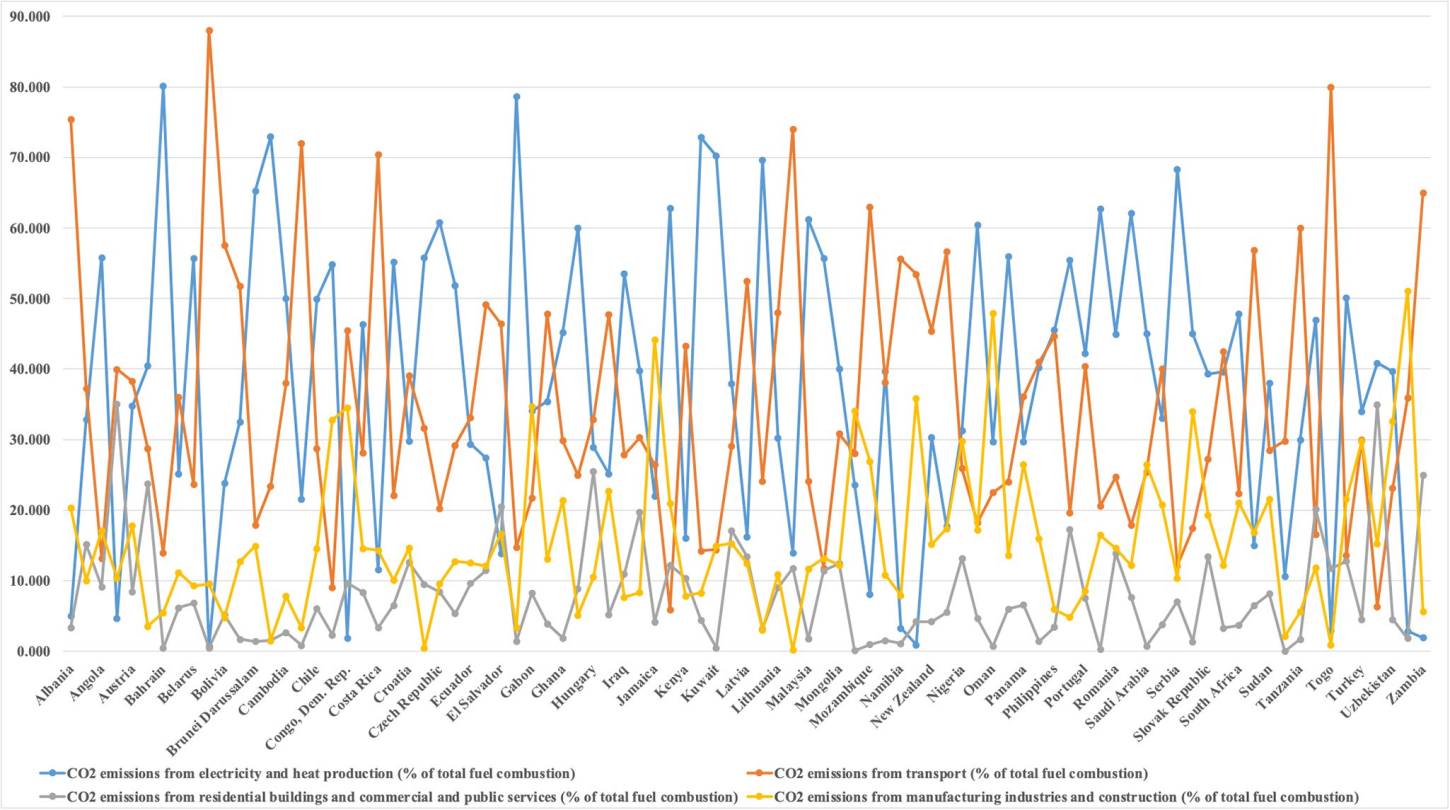
CO_2_ emissions from different sectors of the economies in 2021.

The changes in climate caused by human activities plays a significant influence on ecological business settings. To a large extent, scientists and environmentalists agree that continued increases in carbon emissions (CO_2_) are one of the most serious environmental threats, as they are raising temperatures and growing weather irregularities, and will eventually affect long-term climate fluctuations around the world. CO_2_ emissions are causing widespread alarm due to their dire consequences for the ecosystem and all life on the planet. CO_2_ emissions are a concern for the whole globe, not just one country since no country can face such global issues alone. As a result, tackling environmental issues requires a worldwide collective effort [2, 3]. CO_2_ emissions are a massive threat to a sustainable ecosystem. An expansion in the frequency and severity of severe weather events, increasing sea levels, and biodiversity shifts significantly contribute to human caused climate change that is due to CO_2_ emissions [4, 5]. Particularly, in the case of BRI countries, Fig 1 shows the carbon emissions from different sectors of the economies percentage of total fuel combustion in years 2021.

**Fig 2:**
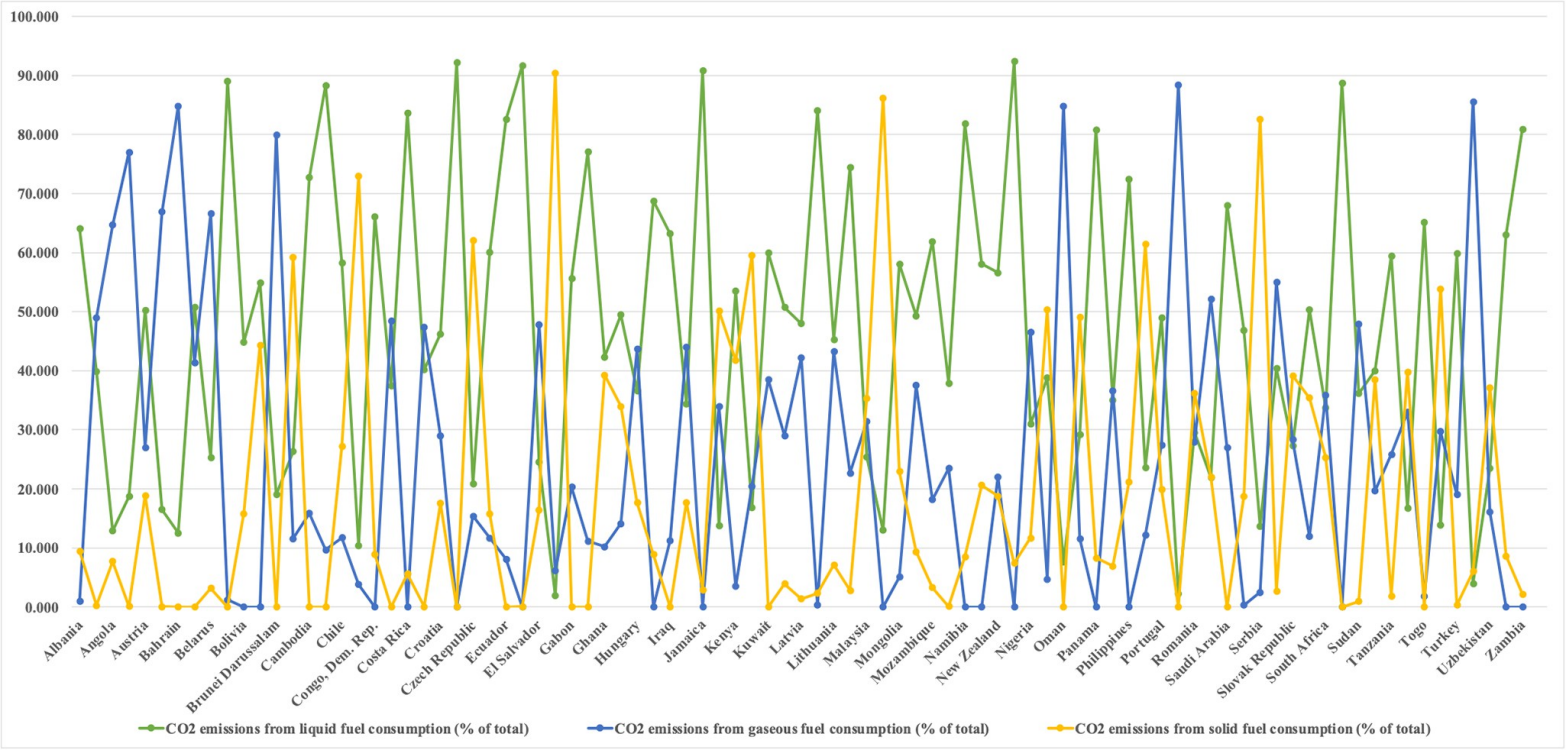
CO_2_ emissions from different energy production sources in 2021.

The major source of these emissions is fossil fuels combustion particularly from coal, oil, and gas. The relative price, technical efficacy, accessibility, and energy content of fossil fuels keep the country using fossil fuels at the largest percentage of worldwide energy consumption. [1, 6] they concluded from their research that using fossil fuel energy increases CO_2_ emissions. Scholars and policymakers are concerned about the environment, and they are examining the relationship between specific CO_2_ emissions and a sustainable environment. [7] found in their research that to reduce CO_2_ emissions, one approach is the transition from using coal, a dirty fuel, to cleaner alternatives such as renewable energy. Fig 2 explains that carbon emission in BRI countries from different source of energy production such as fossil fuels in the form of liquid, solid and gaseous, in year 2021 (percentage of total fuel consumption). This indicates liquid form of fossil fuel has the highest level of energy production source. However, the renewable energy sources such as biodiesel and ethanol, there emissions may balance by trees growth for environmental suitability. While there has been a surge in public awareness of usage of renewable energy and the inclusion of "decarbonization" policies in policy frameworks, these policies have only been implemented in a few countries throughout the world.

**Fig 3:**
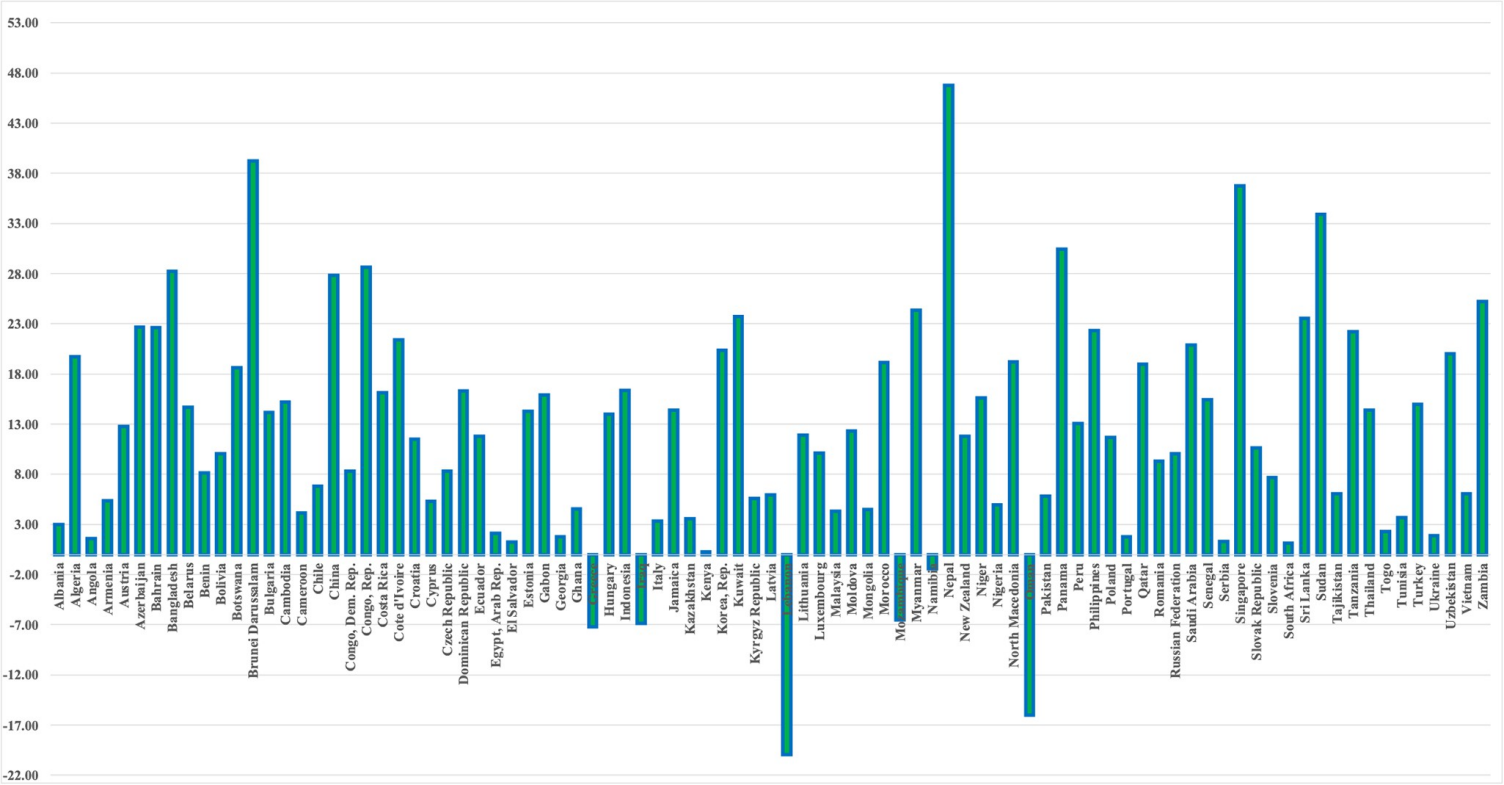
Environmental sustainability.

There is need to understand the linkage among environmental sustainability, disaggregated emissions, renewable energy, and energy efficiency in term of environmental sustainability. Environmental sustainability is a collection of environmentally friendly characteristics and traits [8–11]. The preservation of environmental quality and the attainment of environmental sustainability is the subject of increasing research. Studies by [12–17] emphasized on the association between CO_2_ emissions and environmental sustainability for the different samples and used a variety of econometrics approaches. In the case of environmental sustainability and carbon dioxide emission nexus, the causality can be categorized into three types: unidirectional, bi-directional, and the absence of causality (no causality). As time goes on, there are several ways to interpret the connection between economic growth and carbon dioxide emissions. The one-way relationship between GDP growth and carbon dioxide emissions, meaning that as the economy expands, so do the emissions. Conversely, there is a two-way relationship between economic expansion and emissions. Growth in the economy may increase emissions, but increased emissions can also have an impact on growth in the economy. This reciprocal relationship is known as feedback causation. Even with this connection, it’s crucial to remember that there isn’t a direct correlation indicating that economic activity and environmental quality are wholly unrelated. Despite their connection, their relationship’s precise nature is not always clear-cut and fluctuates based on a variety of circumstances [4, 18–20]. Fig 3 shows the environmental sustainability measured by Adjusted net savings, excluding particulate emission damage (percentage of GNI) in year 2021.

Various fossil fuels emit varying levels of emissions. Consequently, formulating policy decisions that rely on the causal relationship between economic growth and aggregated emission statistics becomes a complex task. As an illustration, it is observed that the combustion of one megajoule (MJ) of coal results in the emission of 92 grams (g) of CO_2_, while the combustion of one megajoule of oil and natural gas yields 74g and 56g of carbon dioxide, respectively. Therefore, conducting a causal analysis to investigate the correlation between economic growth and disaggregated emission data is expected to yield valuable insights for the formulation of policies aimed at promoting a sustainable and environmentally friendly economy [7, 12–17, 21–32].

In light of the escalating influence of climate change, several scholars, including [33–36] have examined the interconnections between CO_2_, energy consumption, and economic growth at a macroscopic level. In contrast, previous studies conducted by [37–41] have explored the empirical relationships between the disaggregation of carbon dioxide emissions (specifically oil, gas, coal, and electricity), energy consumption, and economic growth at a disaggregated level. The findings corroborated the existence of the environmental Kuznets curve (EKC), a theoretical framework that illustrates the inflection point of carbon emissions in relation to economic development.

The relationships among carbon emissions, energy consumption, and economic growth at a collective level are not well-defined due to the diverse causal impacts of different energy sources on both economic growth and carbon emissions [42–44]. Hence, recent scholarly investigations conducted by [42, 45–48] have directed their attention towards examining the interconnections between carbon emissions, energy consumption, and economic growth at a more detailed level in order to obtain more robust empirical findings.

**Fig 4:**
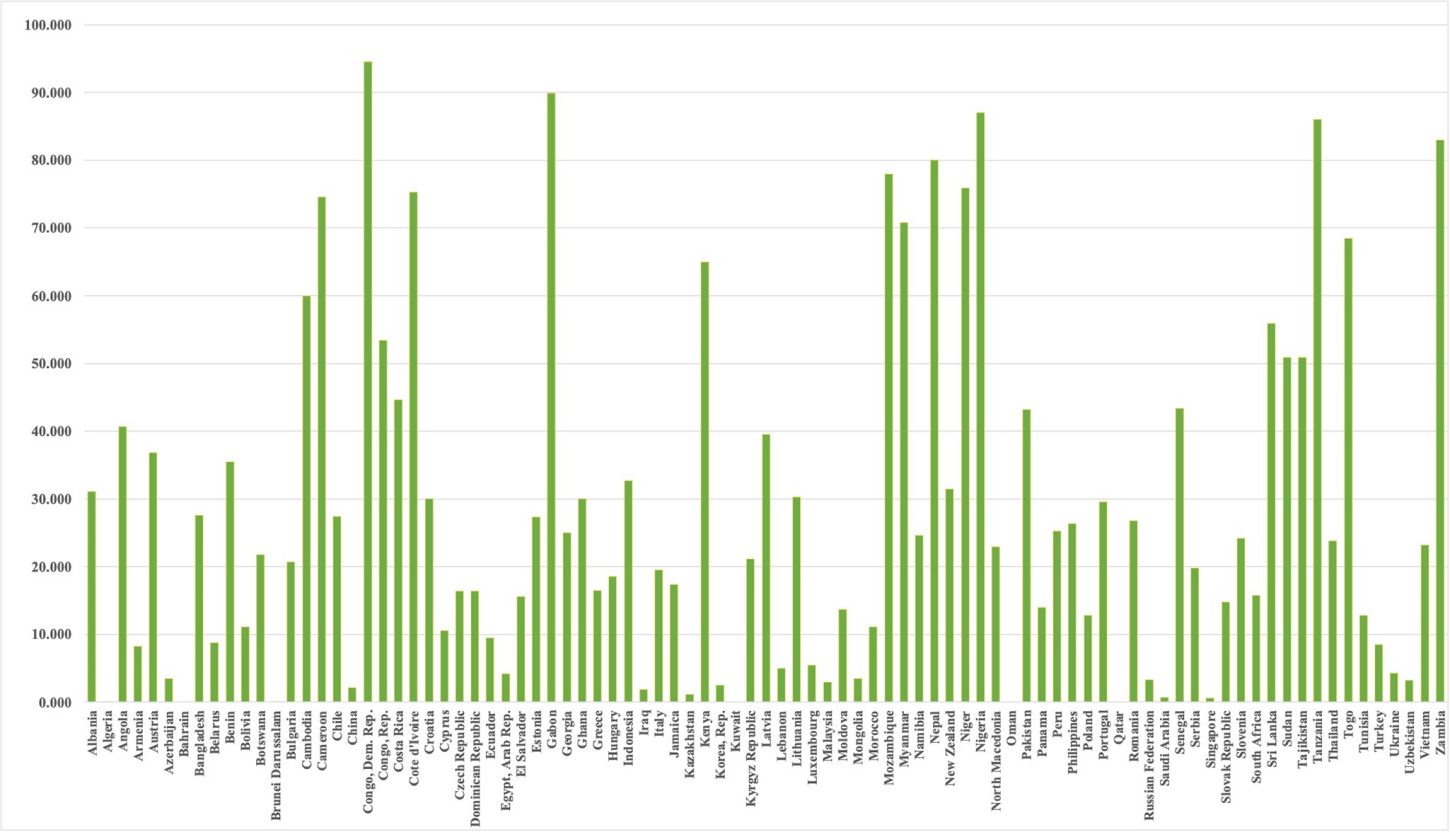
Renewable energy consumption (% of total energy consumption 2021).

[47, 49] noted that previous studies have mostly focused on examining individual energy sources when assessing environmental degradation and energy consumption. The research did not take into account all significant energy sources concurrently for the analysis. This study addresses a research vacuum in the existing literature by examining the relationship between energy consumption, economic growth, and disaggregated carbon emissions (specifically from coal, gas, oil, and electricity) via the lens of the Environmental Kuznets Curve (EKC) hypothesis, as proposed by [18] Comparison between BRI countries using renewable energy is given in Fig 4 and renewable energy consumption measured by percentage of total energy consumption in 2021.

The main idea of this study revolves around exploring the interconnected relationships between various environmental sustainability and energy-related factors within the context of countries participating in the Belt and Road Initiative (BRI). The Belt and Road Initiative is a global infrastructure development strategy initiated by the Chinese government, aiming to enhance economic connectivity across continents. The purpose of this study is to investigate how environmental sustainability, disaggregated emissions, renewable energy utilization, and energy efficiency are interlinked within the BRI countries.

Here are four potential objectives for the study: the first objective is to evaluate the environmental sustainability of BRI countries which involves measuring the extent to which these countries are able to generate economic output while minimizing their environmental impact. This study used indicators such as energy efficiency, renewable energy, to quantify for environmental sustainability. The second objective is to analyse the disaggregated emissions within BRI countries which involves breaking down emissions into various sectors and sources.

The third objective focuses on exploring the adoption and integration of renewable energy sources through assessing the extent to which these countries are incorporating renewable energy and green technologies. This analyses policies, incentives, and investments that promote renewable energy adoption and their impact on environmental sustainability. The fourth objective is to evaluate the energy efficiency measures in place within BRI countries by assessing the effectiveness of policies, technologies, and practices that aim to reduce energy consumption while maintaining or increasing economic output.

Therefore, study aims to provide a comprehensive understanding of the complex interactions between environmental sustainability, disaggregated emissions, renewable energy adoption, and energy efficiency within the context of BRI countries. The rest of this paper is organized as follows: Literature review section presents a brief literature review, methodology section discuss data sources and methodology, results and discussion of the empirical analysis present in results and discussion section, whereas conclusion section describes conclusion.

## Literature review

Several previous studies have examined the linkages between environmental sustainability, disaggregated emissions, renewable energy, and energy efficiency. For instance, [50] investigated the relationship between environmental sustainability and energy consumption in Brazil, finding that improving energy efficiency can enhance environmental performance. [51] conducted a comparative analysis of renewable energy deployment in BRI countries and highlighted the role of policy support in promoting sustainable energy transitions. These studies provide valuable insights into the linkages between these variables, but further research is needed to understand the specific dynamics within the BRI context. Our study builds upon several novel ways. First and foremost, our research attempts to thoroughly assess the environmental sustainability of the BRI by taking into account many factors, including the use of renewable energy sources and energy efficiency. Although other research has addressed related topics, our study integrates these components in a novel way to provide a more comprehensive evaluation. Moreover, our research explores the disaggregated emissions among BRI nations, going beyond the aggregated examination of emissions. Our goal is to provide a comprehensive knowledge of the distinct factors that contribute to the environmental effect in these nations by dissecting emissions into different sectors and sources. This method offers a more focused and in-depth view of emission patterns.

Furthermore, the attention we have paid to energy efficiency measures broadens the scope of our research and offers a more thorough comprehension of the intricate relationships that exist between emissions, environmental sustainability, the adoption of renewable energy sources, and energy efficiency in the context of BRI nations. Our study intends to close the research gap by offering a more thorough and nuanced analysis of the environmental sustainability picture in BRI nations, taking into account energy efficiency measures, the adoption of renewable energy sources, and broken-down emissions in an all-encompassing way. We have carefully noticed and will add the reviewer’s comments about energy transition and inefficient energy consumption from fossil fuels into the literature review in order to further contextualize our work.

[52–55] explained the ambiguous causal association between renewable energy consumption and economic growth. Previous studies consider less of environmental efficiency and deployment of renewable energy resources for reducing carbon emissions. There is need to analyze the association between renewable energy and environmental degradation more rigorously. Because usage of renewable energy sources condenses carbon emissions and enhance environmental quality. Hence, it is vital to establish connection between renewable energy and environmental degradation in the form of disaggregated carbon emissions from different sources and sectors. This will help economies to invest in renewable energy production to create sustainable environment. Few of the researchers had debated on the two-way relationship between renewable energy and environmental degradation. For example, [56] explained the causal relation between hewable energy and carbon emission for 19 countries yearly data from 1984–2007. The empirical results by using [57] expressed that in long run renewable energy consumption lessens carbon emissions. Similarly, [58] used time series data and employed Granger causality test to examine the causal relationship between carbon emissions, renewable energy consumption and nuclear energy. Their results reveal that renewable energy has a substantial impact on carbon emanations.

[59] applied quantile regression on 30 countries data from 1980–2014 and found via empirical reassert that renewable energy play a significant role in mitigating CO_2_ emissions. They established that there is limited effect of usage of more renewable energy to decrease environmental degradation in high emission economies such as USA and China. [60] stressed on government subsidies can help to mitigate environmental degradation. [61] scrutinised the association between carbon emissions and renewable energy through desegrated source such as biomass, solar, wind, wood, biofuel, water, geothermal by for data of USA for the period of 1995–2017. The results presented that usage of different renewable energy sources supports in combating environmental degradation. In lower and upper quantiles, there was significant relation between renewable energy and carbon emissions. However, in middle quantile the effect of renewable energy develops more significant and stronger impact. The findings of the study recommend US to consider renewable energy policies to combat carbon emissions.

[62, 63] studied the impact of energy consumption on environmental quality extensively. These studies have consistently demonstrated that energy consumption is a significant driver of CO_2_ emissions. [26, 62] also explained the similar results. In their study, [64] examine the correlation between energy consumption and CO_2_ emissions across the G-7 nations and suggested that CO_2_ emissions have a direct impact on the adoption of clean energy sources. [65] investigated the relationship between energy consumption, economic expansion, and CO_2_ emissions in the United Kingdom (UK).

Employing the autoregressive distributed lag (ARDL) model, the authors observe that energy policy certainty has resulted in a reduction of CO_2_ emissions in the UK between the years 1985 and 2017. [19] employ the fully modified least squares (FMOLS) approach to examine the relationship between natural resource dependence and the orthodox Environmental Kuznets Curve (EKC) hypothesis. Their findings indicate that economies reliant on natural resources do not conform to the expected trajectory outlined by the EKC hypothesis. According to [66], the presence of ample natural resources in South Africa has been found to be a contributing factor to pollution. However, other studies such as [67, 68] have provided evidence supporting the notion that plentiful natural resources lead to a reduction in CO_2_ emissions.

According to [69] deforestation is identified as the second most significant human-caused contributor of carbon dioxide emissions to the atmosphere, following fossil fuel combustion. According to [70], it has been shown that forests are of great importance in the pursuit of goals pertaining to climate mitigation and adaptation. Based on the findings of the World Health Organization (2012), it was determined that a total of 12.6 million individuals, accounting for 23% of all fatalities recorded during that particular year, succumbed to mortality as a direct consequence of unfavourable living conditions and exposure to hazardous work environments. According to [71] the mortality rates in the Commonwealth of Independent States (CIS) region are significantly influenced by the presence of fossil fuels and the emissions of carbon dioxide. In general, the increase in carbon dioxide emissions.

It has been become a challenge for the economies to maintain balance between financial economic growth and environmental degradation for desire environmental sustainability. [12] used ARDL to study empirical relationship among energy, globalization and income to achieve the environmental sustainability. The study initiates that energy consumption and globalization upsurges the carbon emissions in long-run as well as in short-run. The study suggested that balancing in environmental policies can help economics to sustain.

## Methodology

The prices of energy from fossil fuels and usage of efficient energy cause energy transition among BRI economics. Many countries in Belt and Road Initiative have higher emission levels which is associated with inefficient energy usage [4, 72]. The development of renewable energy resources is a way to enhance social and economic growth for environmental sustainability in BRI region. Energy from renewable resources being as cost competitive and efficient which reduce the imports of fossil fuels.

We confirmed that this manuscript has not been published elsewhere and is not under consideration by another journal. Ethical approval and informed consent do not apply to this study.

### Data

This study utilises annual the data of the BRI countries for the period of 2000–2021. The model uses the variable of Environmental Sustainability (ES) (Adjusted net savings, excluding particulate emission damage (current US$)) as dependent variable. We followed the research done by [73–76] to take this measure for ES. The explanatory variables such as energy efficiency (GDP per unit of energy usage), renewable energy (percentage share of renewables in the total energy consumption) and disaggregated carbon emissions from all over source (the kilotons of the burning from oil, coal, and gas) and sectoral division (Heat and power, Transportation, Residential, Manufacturing). The data has been sourced from World Bank Development Indicators (WDI) from 2000 to 2021 for BRI countries. Variable description, abbreviations and the sources are given below in Table 1.

**Table 1:** Variables description.

Variables	Description	Abbreviations	Source
Environmental Sustainability	Adjusted net savings, excluding particulate emission damage (current US$)	ES	WDI
Energy Efficiency	GDP per unit of energy usage	EE	WDI
Renewable Energy	Percentage share of renewables in the total energy consumption	RE	WDI
Carbon emissions	Source wise (per capita from oil, coal, and gas (% of total fuel consumption)), Sector wise (Heat and power, Transportation, Residential, Manufacturing (% of total fuel combustion))	CO_2_	WDI

### Model estimation

There are plenty of research offered on the relationship among renewable and non-renewable energy consumption and environmental degradation due to carbon emissions [77–82]. The present study employed the nexus among environmental sustainability, energy efficiency, renewable energy, and environmental degradation. The estimate models of this study are given below:

$$\begin{array}{l} Environmental\;Sustainability_{i,t}=\alpha +\beta_{1}Energy\;Efficiency_{i,t}+\beta_{2}Renewable\;Energy_{i,t}+\beta_{3}CO_{2}\\ from\;Oil_{i,t}+\beta_{4}CO_{2}from\;Coal_{i,t}+\beta_{5}CO_{2}\;from\;Gas_{i,t}+\beta_{6}TotalCO_{2i,t}+\varepsilon_{i,t} \end{array}$$ (1)

$$\begin{array}{l} Environmental\;Sustainability_{i,t}=\alpha +\beta_{1}Energy\;Efficiency_{i,t}+\beta_{2}Renewable\;Energy_{i,t}+\\ \beta_{3}CO_{2}EH_{i,t}+\beta_{4}CO_{2}Tr_{i,t}+\beta_{5}CO_{2}R_{i,t}+\beta_{6}CO_{2}M_{i,t}+\beta_{7}CO_{2}Other_{i,t}+\varepsilon_{i,t} \end{array}$$ (2)

The study employs logarithmic transformation with constant variance where α embodies the intercept term, the partial slope coefficients od variable is denoted by βs, and εi, t is the stochastic term. ARDL (Autoregressive Distributed Lag) are not capable of controlling bias if there is correlation exist in the data between mean-differenced variable and white noise term. Is such situation, combination of PMG estimator by [83] and ARDL model offer a way to handle challenge [84]. This study utilizes PMG-ARDL method similar to [84] study. This study employs three distinct routes for the empirical analysis:

1. The stationarity test is conducted using the Fisher Augmented Dickey-Fuller (ADF) test and the unit root test proposed by [83]. The analysis of cointegration and the estimation of long-run regression are advanced by [83].
2. The application of [85] causality analysis methodology.

However, an initial calculation of descriptive statistics and correlation analysis was performed.

## Results and discussion

The results of the study divided into two sections, sources of CO_2_ emissions and sector-wise CO_2_ emission. Table 2 reported the summary statistics of the study in terms of overall sources of CO_2_ emissions. This is required to determine the fundamental measure of the variables’ central tendency and dispersion, as well as how they performed across the study period (2000–2020). Table 2 demonstrated that carbon dioxide emissions ranged from a minimum of 0 kt to a maximum of 93160 kt, 100000 kt, and 91350 kt during the period from various sources, including gas, liquid, and solid/coal, respectively. All variables under consideration are positively skewed. Given the inability to reject the null hypothesis of normality, the current investigation is undertaken with a sample size panel of 288 observations with all series not normally distributed with the exception of carbon dioxide, which is regularly distributed.

**Table 2:** Summary statistics.

	ES	EE	RE	CO_2_ GFC	CO_2_LFC	CO_2_SFC
**Mean**	10.01	9.46	30.34	22.36	50.93	17.62
**Median**	9.10	8.96	21.62	15.27	50.53	6.78
**Maximum**	46.76	27.76	98.34	93.16	100.00	91.35
**Minimum**	-42.89	0.64	0.00	0.00	1.92	0.00
**Std. Dev.**	10.80	4.25	28.18	22.84	25.02	22.73
**Skewness**	0.08	0.62	0.89	0.96	0.09	1.41
**Kurtosis**	4.20	3.52	2.58	3.15	1.97	4.11
**Jarque-Bera**	114.63	139.29	258.53	288.17	85.59	717.70
**Probability**	0.00	0.00	0.00	0.00	0.00	0.00
**Observations**	1868.00	1868.00	1868.00	1868.00	1868.00	1868.00

NB: *** indicates that the null hypothesis was rejected at the 1% level of significance.

In addition to doing a summary statistical analysis, we conducted a correlation matrix analysis to examine the relationship between the variables of interest, as presented in Table 3. During the sampled time, a noteworthy and statistically significant correlation was observed between environmental sustainability and efficiency. However, reviewable energy and CO2 emission from coal has negative impact on environmental sustainability. A negative statistical relationship between renewable energy usage and carbon dioxide emissions is discovered, which is desired for the investigated sample. This suggests that renewable energy sources help to reduce CO2 emissions.

**Table 3:** Correlation matrix analysis.

	**ES**	**EE**	**RE**	**CO**_**2**_ **GFC**	**CO**_**2**_ **LFC**	**CO**_**2**_ **SFC**
**ES**	1.000					
**t-stat**	-----					
**Prob.**	-----					
**EE**	0.116	1.000				
**t-stat**	5.062	-----				
**Prob.**	0.000	-----				
**RE**	-0.035	-0.135	1.000			
**t-stat**	-1.511	-5.867	-----			
**Prob.**	0.131	0.000	-----			
**CO**_**2**_ **GFC**	0.007	-0.123	-0.296	1.000		
**t-stat**	0.304	-5.362	-13.374	-----		
**Prob.**	0.761	0.000	0.000	-----		
**CO**_**2**_ **LFC**	0.068	0.307	0.346	-0.597	1.000	
**t-stat**	2.948	13.917	15.920	-32.106	-----	
**Prob.**	0.003	0.000	0.000	0.000	-----	
**CO**_**2**_ **SFC**	-0.081	-0.194	-0.221	-0.259	-0.556	1.000
**t-stat**	-3.523	-8.564	-9.783	-11.561	-28.909	-----
**Prob.**	0.000	0.000	0.000	0.000	0.000	-----

A correlation matrix analysis is employed in order to investigate the relationship between the variables being examined. Please note that in this context, the symbols ***, **, and * denote rejection levels of 0.01, 0.05, and 0.10, respectively.

It is worth noting that estimate of correlation coefficients alone is insufficient to validate any outcome. Based on the preceding assumption, this study will undertake more reliable and consistent econometric analysis to either confirm the objectives of the study. The stationarity test is used in econometrics analysis to avoid the spurious regression trap. Table 4 shows the unit root analysis, and we found that all variables are stational at the level. As a result of the ADF-Fisher unit root test and the Pesaran Shin unit root test, the general conclusion is that all series are mixed order integrated.

**Table 4:** Unit-root test.

	ADF-FIsher	Im. Pesaran Shin
	Level	Level
**ES**	242.04***	-3.852***
**EE**	268.33***	-5.223***
**RE**	250.79***	-5.007***
**CO**_**2**_ **GFC**	215.03***	-4.241***
**CO**_**2**_ **LFC**	300.25***	-6.406***
**CO**_**2**_ **SFC**	246.71***	-5.333***

Please note that the symbols *** denote a significance level of 0.01 for rejection. The inclusion of a stationarity test is crucial in econometric analysis as it serves to mitigate the risk of falling into the spurious regression trap. It is noted that all variables of interest exhibit first difference stationarity, with the exception of natural resource rent, which remains stationary even at a significance level of 1%. The symbol ***, in this context, denotes a significance threshold of 0.01 for rejecting the null hypothesis.

Following that, we looked at the variables’ long-term association. The Kao residual cointegration test directs that there is a long-run relationship between environmental sustainability, energy efficiency, carbon dioxide emissions, and renewable energy over the time period studied (Table 5).

**Table 5:** Pooled mean group with dynamic autoregressive lag [PMG ARDL (1, 1, 1, 1, 1)].

Model: ES = f (EE, RE, CO_2_GFC, CO_2_LFC, CO2SFC)				
Variables	Coefficient	Std. error	t-statistic	Prob.
**Long run**				
**EE**	1.1427***	0.2313	4.940	0.0000
**RE**	0.0740***	0.0252	2.9410	0.0036
**CO**_**2**_ **GFC**	−0.2234***	0.0527	−4.2398	0.0000
**CO**_**2**_ **LFC**	0.8819***	0.1589	5.5469	0.0000
**CO**_**2**_ **SFC**	0.7341***	0.1087	6.7543	0.0000
**Short run**				
**ECT(-1)**	−0.0700***	0.0241	−2.9041	0.0041
**EE**	0.0336	0.1242	0.2709	0.7101
**RE**	−0.0007	0.0069	−0.0980	0.9220
**CO**_**2**_ **GFC**	−0.1733***	0.0674	−2.5713	0.0065
**CO**_**2**_ **LFC**	1.1228***	0.0931	12.0575	0.0000
**CO**_**2**_ **SFC**	1.0035***	0.0843	11.9045	0.0000
**Constant**	−0.4772***	0.1575	−3.0282	0.0028
**Kao residual cointegration test**				
**ADF**			-3.417	0.0003
**Residual variance**			17.451	
**HAC Variance**			13.533	

Note: the total number of observations in this study is 288. The information criterion used to evaluate the model is the Akaike information criterion (AIC). Based on the AIC, the maximum lag chosen for the model is 1, since it is considered the most parsimonious option.

Please notice that the symbols *** represent a rejection level of 0.01, respectively.

As a result, we evaluate the extent of cointegration given in Table 5 using PMG-ARDL. The results reveal that the contribution of additional regressors to their equilibrium path leads in a robust estimate with a convergence speed of 7%. The current analysis finds a considerable positive association between environmental sustainability and energy efficiency in the long run. One percent gain in energy efficiency using green technology results in a corresponding 1.14% increase in environmental sustainability over time. Similarly, renewable energy has also positive impact on environmental sustainability. Moreover, carbon emissions in gaseous state have negative relation with environmental suitability, indicating 1 percent increase in carbon emissions lead to decrease in environmental sustainability by 0.22 percent.

Here results suggest to environmental administrators and policymakers in the region should reduce CO_2_ emissions for the sake of sustainability. Similarly, although not statistically significant, there is a positive linear relationship between environmental sustainability and energy efficiency. In the sampled region, in both the long and short term, environmental sustainability has a positive correlation with energy efficiency, and negative relation with carbon emissions in form of vaporous. However, environmental sustainability has positive relation with renewable energy in long run and negative relationship in short run. This is desirable because exploration for natural resources directly stimulates economic growth, which in turn increases CO_2_ emissions.

This result is of interest to energy and environmental economists because a one percent increase in the consumption of renewable energy sources leads to a 0.07 percent in short-term and a 07 percent increase in long-term in environmental sustainability among BRI countries This outcome is commendable, and one possible explanation is that the many BRI nations have signed the Kyoto Protocol to reduce CO_2_ emissions. This perspective is corroborated by recent research conducted by [7, 86].

The causality test of the Dumitrescu and Hurlin panel is ultimately reported in Table 6. According to [85] the panel causality test utilized in this study permits the investigation of Granger non-causality from independent variable to dependent variable in a heterogeneous panel context. There is a relationship in both directions between environmental sustainability and energy efficiency. This indicates that industrial activities which includes green technologies stimulate energy efficiency along with economic growth in BRI countries. In other words, this could be a mechanism of feedback between environmental sustainability and energy efficiency in BRI nations. To mitigate climate change and its consequences, a structural transition from an energy- and carbon-intensive economy to a decarbonized economy and services is required [87].

**Table 6:** Dumitrescu and Hurlin panel causality test.

Null hypothesis	W-stat	Prob.	Causality
**EE Dose not homogenously cause ES**	5.40844	0.0000	ES ↔ EE
**ES Dose not homogenously cause EE**	4.13127	0.0000	
**RE Dose not homogenously cause ES**	5.15238	0.0000	ES ↔ RE
**ES Dose not homogenously cause RE**	3.08804	0.0050	
**CO**_**2**_ **GFC Dose not homogenously cause ES**	3.77974	0.0003	ES ↔ CO_2_GFC
**ES Dose not homogenously cause CO**_**2**_ **GFC**	2.61389	0.0000	
**CO**_**2**_ **LFC Dose not homogenously cause ES**	3.61104	0.0000	ES ↔ CO_2_LFC
**ES Dose not homogenously cause CO**_**2**_ **LFC**	7.17121	0.0000	
**CO**_**2**_ **SFC Dose not homogenously cause ES**	3.32310	0.0469	ES ↔ CO_2_SFC
**ES Dose not homogenously cause CO**_**2**_ **SFC**	5.42488	0.0537	
**EE Dose not homogenously cause RE**	5.40608	0.3133	RE → EE
**RE Dose not homogenously cause EE**	3.44934	0.0103	
**RE Dose not homogenously cause CO**_**2**_ **GFC**	2.06495	0.0155	RE ↔ CO_2_GFC
**CO**_**2**_ **GFC Dose not homogenously cause RE**	3.40231	0.0000	
**RE Dose not homogenously cause CO**_**2**_ **LFC**	8.98250	0.0000	RE ↔ CO_2_LFC
**CO**_**2**_ **LFC Dose not homogenously cause RE**	1.64407	0.0028	
**RE Dose not homogenously cause CO**_**2**_ **SFC**	4.80385	0.0041	RE → CO_2_SFC
**CO**_**2**_ **SFC Dose not homogenously cause RE**	5.53748	0.7101	
**EE Dose not homogenously cause CO**_**2**_ **GFC**	1.91565	0.9220	EE → CO_2_GFC
**CO**_**2**_ **GFC Dose not homogenously cause EE**	3.86809	0.0065	
**EE Dose not homogenously cause CO**_**2**_ **LFC**	7.32804	0.0000	EE ↔ CO_2_LFC
**CO**_**2**_ **LFC Dose not homogenously cause EE**	1.42762	0.0000	
**EE Dose not homogenously cause CO**_**2**_ **SFC**	4.71698	0.0028	EE ↔ CO_2_SFC
**CO**_**2**_ **SFC Dose not homogenously cause EE**	5.06321	0.0000	
**CO**_**2**_ **GFC Dose not homogenously cause CO**_**2**_ **LFC**	2.57876	0.0036	CO_2_LFC ↔ CO_2_GFC
**CO**_**2**_ **LFC Dose not homogenously cause CO**_**2**_ **GFC**	2.70932	0.0000	
**CO**_**2**_ **GFC Dose not homogenously cause CO**_**2**_ **SFC**	2.58091	0.0000	CO_2_SFC ↔ CO_2_GFC
**CO**_**2**_ **SFC Dose not homogenously cause CO**_**2**_ **GFC**	2.47556	0.0000	
**CO**_**2**_ **SFC Dose not homogenously cause CO**_**2**_ **LFC**	3.01859	0.0000	CO_2_SFC ↔ CO_2_LFC
**CO**_**2**_ **LFC Dose not homogenously cause CO**_**2**_ **SFC**	5.61676	0.0000	

Note: The symbols ***, **, and * represent rejection levels of 0.01, 0.05, and 0.10, respectively. The symbols ≠, →, and ↔ are used to denote different types of causality relationships. Specifically, ≠ represents No Granger causality, → represents one-way causality, and ↔ represents bi-directional causation.

There is also a feedback mechanism between environmental sustainability and energy efficiency, between environmental sustainability and renewable energy, between environmental sustainability and carbon emission in all forms (Gaseous, Liquid, Solid). The renewable energy impacts environmental sustainability, carbon emissions from gaseous and solid states in one way. However, renewable energy has two-way impact on carbon emission from liquids state. When fossil fuel energy consumption accelerates carbon dioxide emissions, resulting in extreme climate change events, nations increase the proportion of renewable energy technologies in their energy balance. In a different scenario, both nonrenewable and renewable energy contribute to economic expansion, and vice versa.

[88] observed that renewable energy technologies offer several advantages when compared to fossil fuel energy technologies. These advantages include a reduction in energy imports, diversity of energy supply, decreased exposure to price volatility, and the potential to enhance energy security. Natural resource rent plays a pivotal role in the majority of global economies. This study aims to investigate the causal relationship between natural resource rent and economic growth. The investigation and utilization of a country’s natural resources contribute to the growth and development of its economy. However, as indicated by [20], the excessive utilization of existing natural resources has the effect of diminishing a nation’s biocapacity, which refers to the capacity of natural resources to regenerate, and concurrently amplifies its ecological footprint, leading to the emergence of an ecological deficit. The shift from conventional technologies, which heavily rely on natural resources, to contemporary technologies that integrate recycling, reuse, innovation, value addition, and artificial resources as substitutes for natural resources, has the potential to bolster economic progress while mitigating environmental deterioration. Within the European Union, there exists a symbiotic relationship between nonrenewable and renewable energy sources. The successful integration of either energy source into a country’s energy mix is contingent upon the presence and utilization of the other, in order to achieve sustainable production and consumption. Renewable energy systems often encounter challenges related to intermittency and stability, while fossil fuel energy methods are associated with carbon-intensive issues.

Hence, [84] put up a strategic amalgamation of these two energy sources as a means to attain widespread availability of contemporary and cost-effective energy services, while concurrently addressing the repercussions of climate change. The research findings revealed a unidirectional correlation between the rent derived from natural resources and the emissions of carbon dioxide, as well as between the rent derived from natural resources and the utilization of both renewable and nonrenewable energy sources. Hence, the presence of natural resources has a significant role in influencing the utilization of both renewable and nonrenewable sources of energy. On the other hand, within an economy that heavily relies on natural resources, an excessive dependence on revenue generated from these resources leads to the deterioration of the environment. In order to achieve a sustainable environment, it is imperative to implement diversification and structural changes in economic development.

### Sector-wise CO_2_ emission

This study chooses four major sectors in term on high energy consumption which ultimately have impact on environment sustainability. The sector-wise CO_2_ emission includes carbon emission from heat and power sector, transportation sector, residential sector and manufacturing sector and other sectors. Table 7 reported the summary statistics of the study in terms of sector wise CO_2_ emissions. This is required to determine the fundamental measure of the variables’ central tendency and dispersion, as well as how they performed across the study period (2000–2020). Table 7 demonstrated that carbon dioxide from heat and power sector produced emission of 88700 kt, 12242kt from transportation sector, 551500kt from manufacturing sector, 869600kt from other sectors. All variables under consideration are positively skewed.

**Table 7:** Summary statistics.

	ES	EE	RE	CO_2_EH	CO_2_Tr	CO_2_Res	CO_2_Other	CO_2_M
**Mean**	10.01	9.45	30.33	37.98	33.08	8.97	4.15	16.03
**Median**	9.09	8.95	21.61	38.68	28.76	7.96	1.82	14.50
**Maximum**	46.76	27.76	98.34	88.70	122.42	39.66	86.96	55.15
**Minimum**	-42.89	0.64	0.00	-5.53	1.83	0.00	-0.23	0.00
**Std. Dev.**	10.80	4.25	28.18	19.98	18.65	7.03	9.03	8.91
**Skewness**	0.08	0.62	0.89	-0.10	1.05	1.35	6.13	0.93
**Kurtosis**	4.21	3.51	2.58	2.53	4.17	5.50	47.57	4.09
**Jarque-Bera**	115.11	139.35	259.02	20.31	448.30	1052.29	166413.50	361.00
**Probability**	0.00	0.00	0.00	0.00	0.00	0.00	0.00	0.00
**Observations**	1869.00	1869.00	1869.00	1869.00	1869.00	1869.00	1869.00	1869.00

In addition to the summary statistical analysis, as shown in Table 8, we conducted a correlation matrix analysis to evaluate the relationship between the variables under discussion. A positive and significant relationship observed between environmental sustainability and efficiency for the sampled period. However, reviewable energy has negatively impact on environmental sustainability. A negative statistical relationship between renewable energy usage and carbon dioxide emissions for heat and power sector and residentials sector is discovered, which is desired for the investigated sample. In contrast, environmental sustainability has positive relation with carbon emission from manufacturing and transportation sector.

**Table 8:** Correlation matrix analysis.

	**ES**	**EE**	**RE**	**CO**_**2**_ **EH**	**CO**_**2**_ **Tr**	**CO**_**2**_ **Res**	**CO**_**2**_ **Other**	**CO**_**2**_ **M**
**ES**	1.000							
**t-stat**	-----							
**Prob.**	-----							
**EE**	0.116	1.000						
**t-stat**	5.066	-----						
**Prob.**	0.000	-----						
**RE**	-0.035	-0.134	1.000					
**t-stat**	-1.509	-5.844	-----					
**Prob.**	0.132	0.000	-----					
**CO**_**2**_ **EH**	-0.008	-0.100	-0.575	1.000				
**t-stat**	-0.346	-4.364	-30.330	-----				
**Prob.**	0.730	0.000	0.000	-----				
**CO**_**2**_ **Tr**	0.052	0.175	0.510	-0.781	1.000			
**t-stat**	2.260	7.675	25.601	-54.088	-----			
**Prob.**	0.024	0.000	0.000	0.000	-----			
**CO**_**2**_ **Res**	-0.151	-0.089	0.064	-0.223	-0.053	1.000		
**t-stat**	-6.586	-3.876	2.770	-9.878	-2.304	-----		
**Prob.**	0.000	0.000	0.006	0.000	0.021	-----		
**CO**_**2**_ **Other**	-0.070	0.016	0.156	-0.286	-0.045	-0.120	1.000	
**t-stat**	-3.045	0.711	6.827	-12.907	-1.936	-5.243	-----	
**Prob.**	0.002	0.477	0.000	0.000	0.053	0.000	-----	
**CO**_**2**_ **M**	0.100	-0.095	0.028	-0.173	-0.203	-0.068	-0.150	1.000
**t-stat**	4.357	-4.112	1.212	-7.590	-8.952	-2.954	-6.564	-----
**Prob.**	0.000	0.000	0.226	0.000	0.000	0.003	0.000	-----

We conduct a correlation matrix analysis to explore the relationship between the variables under review.

Note: ***, **, * represents 0.01, 0.05 and 0.10 rejection level respectively.

It is important to highlight that relying solely on the estimation of correlation coefficients is inadequate for validating any outcome. This study aims to conduct a more robust and consistent econometric analysis in order to validate the objectives of the study. The utilization of the stationarity test in econometric analysis serves the purpose of mitigating the risk of falling into the spurious regression trap. Table 9 presents the results of the unit root analysis, indicating that all variables exhibit stationarity at the level. Based on the outcomes of the ADF-Fisher unit root test and the Pesaran Shin unit root test, it can be generally inferred that all series exhibit mixed order integration.

**Table 9:** Unit-root test.

	ADF-FIsher	Im. Pesaran Shin
	Level	Level
**ES**	242.04***	-3.852***
**EE**	268.33***	-5.223***
**RE**	250.79***	-5.007***
**CO**_**2**_ **EH**	393.503***	-9.517***
**CO**_**2**_ **Tr**	311.309***	-6.588***
**CO**_**2**_ **Res**	346.141***	-7.842***
**CO**_**2**_ **Other**	263.337***	5.611***
**CO**_**2**_ **M**	344.382***	-7.952***

***, **, * represents 0.01, 0.05 and 0.10 rejection level respectively.

Subsequently, an examination was conducted regarding the enduring correlation between the variables. The findings of the Kao residual cointegration test indicate the presence of a significant and enduring association between environmental sustainability, energy efficiency, renewable energy, carbon dioxide emissions from four major sectors which are heat and power sector, transportaion, residential and manufacturing sector, throughout the analyzed time period (refer to Table 10).

**Table 10:** Pooled mean group with dynamic autoregressive lag [PMG ARDL (1, 1, 1, 1, 1)].

Model: ES = f (EE, RE, CO_2_GFC, CO_2_LFC, CO_2_SFC)				
Variables	Coefficient	Std. error	t-statistic	Prob.
**Long run**				
**EE**	-1.3787***	0.2280	-6.0457	0.0000
**RE**	-0.8580***	0.0792	-10.823	0.0000
**CO**_**2**_ **EH**	2.9854***	0.4357	6.8507	0.0000
**CO**_**2**_ **Tr**	0.0405***	0.0143	2.8329	0.0050
**CO**_**2**_ **Res**	-0.0964***	0.0263	-3.6572	0.0003
**CO**_**2**_ **Other**	−0.1805***	0.0427	−4.2388	0.0000
**CO**_**2**_ **M**	0.8819***	0.1589	5.5469	0.0000
**Short run**				
**ECT(-1)**	-0.3835***	0.0857	-4.4728	0.0000
**EE**	6.2587**	3.1338	1.9971	0.0469
**RE**	2.0176*	1.0408	1.9385	0.0537
**CO**_**2**_ **EH**	3.1089	3.0770	1.0103	0.3133
**CO**_**2**_ **Tr**	0.0476**	0.0184	2.5875	0.0103
**CO**_**2**_ **Res**	-0.1834**	0.0752	-2.4383	0.0155
**CO**_**2**_ **Other**	9.1844***	1.9496	4.7107	0.0000
**CO**_**2**_ **M**	1.1228***	0.0931	12.0575	0.0000
**Constant**	−0.4772***	0.1575	−3.0282	0.0028
**Kao residual cointegration test**				
**ADF**			-3.416266	0.0003
**Residual variance**			17.59047	
**HAC Variance**			13.28480	

Note: number of observations = 288, information criterion-Akaike information criterion (AIC), maximum lag 1 as suggested by AIC and most parsimonious.

***, **, * represents 0.01, 0.05 and 0.10 rejection level respectively.

As a result, we evaluate the extent of cointegration given in Table 10 using PMG-ARDL. The results reveal that the contribution of additional regressors to their equilibrium path leads in a robust estimate with a convergence speed of 38%. The current analysis finds a considerable negative association among environmental sustainability and energy efficiency and renewable energy and carbon emissions from residential and ithers sectors in the long run, however, these variables have positive relation in short run. Moreover, emission from heat and power sector and transportation sector has positive impact on environmental sustainability.

The findings indicate that it would be advisable for environmental administrators and policymakers in the region to implement measures aimed at reducing CO_2_ emissions from transportation, residential, manufacturing and heat and power sectors, to promote sustainability. Likewise, while lacking statistical significance, a positive linear correlation can be shown among environmental sustainability, renewable energy and energy efficiency, in the long run, but negative relationship in short run. In the examined geographical area, there exists a positive association between environmental sustainability and carbon emission from heat and power sector and transportation sector in both the short and long term. Conversely, there is a negative relationship between environmental sustainability and carbon emissions from residential sector. The desirability of search for natural resources stems from its direct stimulation of economic expansion, hence leading to an increase in CO_2_ emissions.

Table 11 presents the causality test conducted by Dumitrescu and Hurlin on the panel data. According to [85] the utilization of the panel causality test in this study allows for the investigation of Granger non-causality from an independent variable to a dependent variable within a heterogeneous panel framework. There is a reciprocal correlation between environmental sustainability and energy efficiency. This implies that the integration of environmentally friendly technologies in industrial operations holds the capacity to improve energy efficiency and stimulate economic development in nations involved in the Belt and Road Initiative (BRI). This phenomenon has the potential to function as a feedback mechanism that establishes a connection between environmental sustainability and energy efficiency among the nations involved in the Belt and Road Initiative (BRI). To effectively tackle the difficulties presented by climate change and its related consequences, it is crucial to make a transformative transition from an economy that heavily relies on energy and activities that emit high levels of carbon to one that is decarbonized and prioritizes sustainable services [87].

**Table 11:** Dumitrescu and Hurlin panel causality test.

Null hypothesis	W-stat	Prob.	Causality
**EE Dose not homogenously cause ES**	6.70479	0.0003	EE↔ES
**ES Dose not homogenously cause EE**	5.88133	0.0099	
**RE Dose not homogenously cause ES**	6.23646	0.0661	RE↔ES
**ES Dose not homogenously cause RE**	5.15037	0.0022	
**CO**_**2**_ **EH Dose not homogenously cause ES**	4.35225	0.0000	CO_2_EH↔ES
**ES Dose not homogenously cause CO**_**2**_ **EH**	5.67021	0.0011	
**CO**_**2**_ **Tr Dose not homogenously cause ES**	7.00091	0.0003	CO_2_Tr↔ES
**ES Dose not homogenously cause CO**_**2**_ **Tr**	5.23395	0.0099	
**CO**_**2**_ **Res Dose not homogenously cause ES**	4.04051	0.0661	CO_2_Res↔ES
**ES Dose not homogenously cause CO**_**2**_ **Res**	7.30939	0.0022	
**CO**_**2**_ **Other Dose not homogenously cause ES**	7.18047	0.0000	CO_2_Other↔ES
**ES Dose not homogenously cause CO**_**2**_ **Other**	3.70350	0.0079	
**CO**_**2**_ **M Dose not homogenously cause ES**	2.40757	0.1213	CO_2_M≠ES
**ES Dose not homogenously cause CO**_**2**_ **M**	2.68833	0.1537	
**EE Dose not homogenously cause RE**	3.44787	0.0000	EE→RE
**RE Dose not homogenously cause EE**	4.66376	0.2161	RE≠EE
**RE Dose not homogenously cause CO**_**2**_ **EH**	3.67335	0.9714	CO_2_EH ≠RE
**CO**_**2**_ **EH Dose not homogenously cause RE**	3.28118	0.7672	
**RE Dose not homogenously cause CO**_**2**_ **Tr**	4.11830	0.3173	RE≠CO_2_tr
**CO**_**2**_ **Tr Dose not homogenously cause RE**	2.16964	0.0334	CO_2_Tr→RE
**RE Dose not homogenously cause CO**_**2**_ **Res**	3.47627	0.2267	CO_2_Res≠RE
**CO**_**2**_ **Res Dose not homogenously cause RE**	4.00046	0.3978	
**RE Dose not homogenously cause CO**_**2**_ **Other**	4.58291	0.1050	CO_2_Other≠RE
**CO**_**2**_ **Other Dose not homogenously cause RE**	7.75791	0.8536	
**RE Dose not homogenously cause CO**_**2**_ **M**	2.85866	0.3048	RE≠ CO_2_M
**CO**_**2**_ **M Dose not homogenously cause RE**	5.40844	0.0000	CO_2_M→RE
**EE Dose not homogenously cause CO**_**2**_ **EH**	4.13127	0.0000	CO_2_EH↔EE
**CO**_**2**_ **EH Dose not homogenously cause EE**	5.15238	0.0000	
**EE Dose not homogenously cause CO**_**2**_ **Tr**	3.08804	0.0050	CO_2_Tr↔EE
**CO**_**2**_ **Tr Dose not homogenously cause EE**	3.77974	0.0003	
**EE Dose not homogenously cause CO**_**2**_ **Res**	2.61389	0.0000	CO_2_Res↔EE
**CO**_**2**_ **Res Dose not homogenously cause EE**	3.61104	0.0000	
**EE Dose not homogenously cause CO**_**2**_ **Other**	7.17121	0.0000	CO_2_Other↔EE
**CO**_**2**_ **Other Dose not homogenously cause EE**	3.32310	0.0469	
**EE Dose not homogenously cause CO**_**2**_ **M**	5.42488	0.0537	EE→CO_2_M
**CO**_**2**_ **M Dose not homogenously cause EE**	5.40608	0.3133	CO_2_M≠EE
**CO**_**2**_ **EH Dose not homogenously cause CO**_**2**_ **Tr**	3.44934	0.0103	CO_2_EH↔CO_2_Tr
**CO**_**2**_ **Tr Dose not homogenously cause CO**_**2**_ **EH**	2.06495	0.0155	
**CO**_**2**_ **EH Dose not homogenously cause CO**_**2**_ **Res**	3.40231	0.0000	CO_2_Res↔CO_2_EH
**CO**_**2**_ **Res Dose not homogenously cause CO**_**2**_ **EH**	8.98250	0.0000	
**CO**_**2**_ **Res Dose not homogenously cause CO**_**2**_ **Tr**	1.64407	0.0028	CO_2_Res↔CO_2_tr
**CO**_**2**_ **Tr Dose not homogenously cause CO**_**2**_ **Res**	4.80385	0.0041	
**CO**_**2**_ **Other Dose not homogenously cause CO**_**2**_ **EH**	5.53748	0.7101	CO_2_Other≠CO_2_EH
**CO**_**2**_ **EH Dose not homogenously cause CO**_**2**_ **Other**	1.91565	0.9220	
**CO**_**2**_ **Other Dose not homogenously cause CO**_**2**_ **Tr**	3.86809	0.0065	CO_2_Other↔CO_2_Tr
**CO**_**2**_ **Tr Dose not homogenously cause CO**_**2**_ **Other**	7.32804	0.0000	
**CO**_**2**_ **Other Dose not homogenously cause CO**_**2**_ **Res**	1.42762	0.0000	CO_2_Other↔CO_2_Res
**CO**_**2**_ **Res Dose not homogenously cause CO**_**2**_ **Other**	4.71698	0.0028	
**CO**_**2**_ **M Dose not homogenously cause CO**_**2**_ **EH**	5.06321	0.0000	CO_2_M↔CO_2_EH
**CO**_**2**_ **EH Dose not homogenously cause CO**_**2**_ **M**	2.57876	0.0036	
**CO**_**2**_ **M Dose not homogenously cause CO**_**2**_ **Tr**	2.70932	0.0000	CO_2_M↔CO_2_Tr
**CO**_**2**_ **Tr Dose not homogenously cause CO**_**2**_ **M**	2.58091	0.0000	
**CO**_**2**_ **M Dose not homogenously cause CO**_**2**_ **Res**	2.47556	0.0000	CO_2_M↔CO_2_Res
**CO**_**2**_ **Res Dose not homogenously cause CO**_**2**_ **M**	3.01859	0.0000	
**CO**_**2**_ **M Dose not homogenously cause CO**_**2**_ **Other**	5.61676	0.0000	CO_2_M↔CO_2_Other
**CO**_**2**_ **Other Dose not homogenously cause CO**_**2**_ **M**	1.56361	0.0000	

Note: The symbols ***, **, and * represent rejection levels of 0.01, 0.05, and 0.10, respectively. The symbols ≠, →, and ↔ are used to denote different types of causality relationships. Specifically, ≠ represents No Granger causality, → represents one-way causality, and ↔ represents bi-directional causation.

## Conclusion

The study investigated the sources of CO_2_ emissions (overall and sectoral division) and their relationship with environmental sustainability and energy efficiency. The study also conducted a correlation matrix analysis, finding a positive and significant relationship between environmental sustainability and efficiency. However, renewable energy sources help reduce CO_2_ emissions. Moreover, Renewable energy also positively impacts environmental sustainability. The study suggests that environmental administrators and policymakers in the region should reduce CO_2_ emissions for sustainability.

The causality test of the Dumitrescu and Hurlin panel indicates a relationship between environmental sustainability and energy efficiency, suggesting that industrial activities incorporating green technologies stimulate energy efficiency and economic growth in BRI countries. The study also suggests a feedback mechanism between environmental sustainability and energy efficiency, suggesting a need for a structural transition from an energy- and carbon-intensive economy to a decarbonized one.

The study also examined the causality of high energy consumption in four major sectors, including heat and power, transportation, residential, and manufacturing sectors, on carbon emissions. A positive relationship between environmental sustainability and carbon emissions from manufacturing and transportation sectors has been found. And a negative association between environmental sustainability and energy efficiency and renewable energy and carbon emissions from residential and other sectors in the long run. However, these variables have a positive relationship in the short run.

The heat and power and transportation sectors have a positive impact on environmental sustainability. The findings suggest that environmental administrators and policymakers should implement measures to reduce CO_2_ emissions from transportation, residential, manufacturing, and heat and power sectors to promote sustainability. It is necessary to integrate environmentally friendly technologies in operations can improve energy efficiency and stimulate economic development in countries involved in the Belt and Road Initiative (BRI).

This phenomenon can function as a feedback mechanism, establishing a connection between environmental sustainability, renewable energy, carbon emissions and energy efficiency. To effectively tackle climate change, it is crucial to transition from an energy-intensive economy to one that prioritizes sustainable services and decarbonizes, focusing on sustainable practices.

This research contributes valuable insights to policymakers, researchers, and stakeholders concerned with sustainable development and environmental protection in these regions. To achieve meaningful environmental sustainability for current and future generations, we propose that governments and policymakers prioritised greenhouse gas emission reductions balance their energy and environmental conservation and protection policies with their macroeconomic goal.
